# Association Between Serum Lipid Levels and Patients With Primary Angle-Closure Glaucoma in China: A Cross Sectional, Case–Control Study

**DOI:** 10.3389/fmed.2021.618970

**Published:** 2021-02-02

**Authors:** Mingxi Shao, Yingzhu Li, Jisen Teng, Shengjie Li, Wenjun Cao

**Affiliations:** Clinical Laboratory, Eye & Ear Nose Throat (ENT) Hospital, Fudan University, Shanghai, China

**Keywords:** primary angle-closure glaucoma, serum lipid concentration, high-density lipoprotein cholesterol, triglyceride, low-density lipoprotein cholesterol, cholesterol

## Abstract

**Objective:** To evaluate the serum lipid levels of patients with primary angle-closure glaucoma (PACG) and to investigate the relationship between serum lipid levels and PACG.

**Methods:** In this cross-sectional, case–control study, a total of 320 PACG subjects and 242 age- and sex-matched control subjects were recruited. Serum high-density lipoprotein cholesterol (HDL-C), low-density lipoprotein cholesterol (LDL-C), small dense LDL-C (SDLDL-C), triglyceride (TRIG), and cholesterol (CHOL) levels were measured using enzymatic colorimetry. Serum apolipoprotein A (APOA), apolipoprotein B (APOB), apolipoprotein E (APOE), and lipoprotein(a) (LPa) levels were measured by immunoturbidimetry.

**Results:** The serum LDL-C, TRIG, HDL-C, APOE, LPa, CHOL, APOB, and APOA levels were significantly higher (*p* < 0.05) in the PACG group than in the control group. Multiple liner regression analyses revealed that there was a statistically correlation between HDL-C levels and mean deviation MD (*B* = 0.389, *P* = 0.002, 95% confidence interval [CI] = −1.249 to −0.624); LDL-C levels and MD (*B* = 0.190, *P* = 0.019, 95% CI = −5.632 to −1.306); and CHOL levels and MD (*B* = 0.364, *P* = 0.27, 95% CI = −7.727 to −1.839). Logistic regression analyses showed that high serum HDL-C (odds ratio [OR] = 11.01, 95% CI = 5.616–21.587), LDL-C (OR = 1.330, 95% CI = 1.079–1.640), SDLDL-C (OR = 1.007, 95% CI = 1.005–1.008), APOA (OR = 13.621, 95% CI = 7.251–25.591), APOB (OR = 2.243, 95% CI = 1.060–4.732), LPa (OR = 0.999, 95% CI = 0.998–1.00), and CHOL (OR = 1.131, 95% CI = 1.005–1.326) levels were significantly associated with PACG.

**Conclusions:** High serum HDL-C, LDL-C, APOA, APOB, LPa, and CHOL levels were associated with PACG.

## Introduction

Glaucoma is a multifactor-induced progressive optic neuropathy with obvious visual field defects, and is the most common irreversible blinding eye disease in the world (1, 2). By the end of 2020, about 79.6 million people will have glaucoma (3). Primary angle-closure glaucoma (PACG) is the most common type of glaucoma in China; however, the exact pathogenesis is still unclear. Currently, identified risk factors include elevated intraocular pressure (IOP) (4), metabolic abnormalities (5), and family history of glaucoma (6). However, other potential risk factors for PACG may also exist; therefore, exploring these potential factors to develop interventions to reduce the incidence of glaucoma is of the utmost urgency.

Recent epidemiological studies have suggested that hyperlipidemia may be associated with glaucoma, but the findings are contradictory. Newman-Casey et al. (7) found that people with hyperlipidemia had a lower risk of primary open angle glaucoma (POAG) than those without hyperlipidemia. However, one study by Lin et al. (8) showed that there was a positive correlation between hyperlipidemia and the development of glaucoma. Hyperlipidemia would increase the risk of glaucoma, and elevated lipid levels were also considered to be a risk factor for increased IOP. The results of Yilmaz et al. (9) also suggested that the pathogenesis of different types of glaucoma may be related to changes in hyperlipidemia.

At present, there are limited relevant studies to explore the association between lipid levels and PACG. Therefore, we performed a cross-sectional, case–control study to clarify the association between lipid levels and PACG.

## Materials and Methods

### Subjects

The study was approved by the Ethics Committee of the Eye and ENT Hospital of Fudan University, Shanghai, China. It was conducted in accordance with the Declaration of Helsinki. Informed consent was obtained from all subjects. Patients with PACG were consecutively recruited from the Department of Ophthalmology and Visual Science, Eye and ENT Hospital of Fudan University between November 2018 and August 2020.

Control subjects were consecutively recruited from individuals who participated in annual health screenings during the study period. The study clerks conducted face-to-face questionnaire interviews to gather data, such as diabetes, hypertension, height, and body weight.

We used an open source calculator to calculate the minimal required sample size based on the probability of a type I error of alpha at 5% (two-sided), type II error of beta at 20%, the Odds Ratio at 2.5, a sample size ratio of 0.8, the normal population have prevalence of PACG that is about 5% in China. This calculation yielded a sample consisting of at least 272 cases and 218 controls.

### Examination

Medical examinations for all subjects included an electrocardiogram, X-ray, and an assessment of liver function, kidney function, infectious disease, blood pressure, heart rate, and body temperature. All subjects underwent medical examinations at the Eye and ENT Hospital of Fudan University. Each PACG patient also underwent a standardized ophthalmic examination performed by glaucoma specialists. The methods used to perform the ophthalmic examinations have been previously described in detail (10–13).

Laboratory testing was performed at the Department of Clinical Laboratory, Eye and ENT Hospital of Fudan University. Blood samples were obtained in the morning after subjects fasted for 8 h. The tubes were centrifuged for 10 min at 3,000 r/min. Serum high-density lipoprotein cholesterol (HDL-C), low-density lipoprotein cholesterol (LDL-C), small dense LDL-C (SDLDL-C), triglyceride (TRIG), and cholesterol (CHOL) levels were measured by enzymatic colorimetry (Roche Cobas 8000C702, Mannheim, Germany). Serum apolipoprotein A (APOA), apolipoprotein B (APOB), apolipoprotein E (APOE), and lipoprotein(a) (LPa) levels were measured by immunoturbidimetry (Roche Cobas 8000C702, Mannheim, Germany). In order to ensure the accuracy of the detection system, the biochemical analyzer conduct an indoor quality control test every day, and the monthly coefficient of variation was controlled within the range of 3–5%.

### Diagnostic and Inclusion Criteria

#### PACG Subjects

PACG was diagnosed by a glaucoma specialist. Previously described diagnostic criteria were used to diagnose PACG and previously described inclusion criteria were used to select patients (11–13). PACG was diagnosed based on narrow anterior chamber angles with glaucomatous optic neuropathy and corresponding VF loss. VF loss was determined by including a cluster of three or more non-edge contiguous points on the pattern deviation plot, which did not cross the horizontal meridian, and had a probability of <5% of being present in age-matched controls (one of which was <1%); it had an abnormal standard deviation pattern with a *p* < 0.05 occurring in the normal population, and fulfilled the following test reliability criteria: <20% fixation losses, <15% false positives, and/or <15% false negatives. Additionally, PACG was diagnosed in eyes with narrow angles, elevated IOP (IOP > 21 mmHg), having at least 180° of angle-closure obliterating the pigmented segment of the trabecular meshwork, whether synechial, appositional, segmented, or continuous, and in cases where the degree of peripheral anterior synechiae was too extensive to be managed by laser peripheral iridotomy. The inclusion criteria for PACG subjects were as follows: absence of any secondary glaucoma or any other eye disease that could potentially affect visual acuity or the VF, absence of any intraocular surgery within the previous 2 months, absence of any systemic diseases including acute infectious diseases, metabolic syndrome, autoimmune disease, or cancer.

A total of 414 participants were recruited, of whom 94 were later excluded (secondary glaucoma = 60, congenital glaucoma = 12, neovascular glaucoma = 10, cataract = 5, systemic diseases = 5, macular degeneration = 2), leaving a final sample of 320 patients (newly diagnosed PACG patients = 202, referral PACG patients = 118). “Newly diagnosed PACG patients” mean that they reported themselves that they have no treatment experience before coming to our hospital. They were first diagnosed with glaucoma at our hospital.

#### Control Subjects

The normal subjects were recruited from health screenings, and without eye discomfort. Moreover, each normal control subject underwent preliminary ophthalmic examinations, which included refractive status, gonioscopy, and slit-lamp biomicroscopic examination, performed by glaucoma specialists. The inclusion criteria for normal subjects were as follows: absence of any type of glaucoma or any other eye disease that could potentially affect visual acuity or the VF, absence of any intraocular surgery within the previous 2 months, absence of any systemic diseases including acute infectious diseases, metabolic syndrome, autoimmune disease, or cancer. A total of 277 control subjects were recruited, of whom 35 subjects were later excluded from the study based on the inclusion criteria (cataract = 8, cancer = 2, renal disease = 6, liver disease = 4, autoimmune disease = 2, macular degeneration = 3, cardiovascular disease = 4, other diseases = 6), leaving a final sample of 242 control subjects. There were no significant difference of demographics and disease severity (*p* > 0.05) between newly diagnosed PACG patients and referral PACG patients (Supplementary Table 1).

### Statistical Analyses

The analyses were performed using version 13.0 of the Statistical Package for the Social Sciences (SPSS Inc., Chicago, IL, USA). The results are presented as mean ± standard deviation (SD). Normality was assessed using the Kolmogorov–Smirnoff test. The independent Student *t*-test and chi-squared test were used to compare the subject characteristics between the groups. One-way analysis of variance (ANOVA) was used to compare the lipid levels among the three groups. Correlations between lipid levels and ocular parameters were assessed using Pearson correlation analysis. Multivariate linear regression analyses were performed to evaluate the association between lipid levels and ocular parameters. Furthermore, logistic regression analyses were performed to identify the risk factors for PACG. A two-sided *p*-value of <0.05 was considered statistically significant.

## Results

### Characteristics of the Study Patients

A total of 320 subjects with PACG (male = 146, female = 174) and 242 control subjects (male = 111, female = 131) were recruited in this study. There was no statistical difference in the mean age and sex between the PACG and control subjects (*P* > 0.05). The serum LDL-C, TRIG, HDL-C, APOE, LPa, CHOL, APOB, and APOA levels were significantly higher (*p* < 0.05) in the PACG group than in the control group. Table 1 presents a summary of the demographics and serum lipid levels of the PACG and control groups.

**Table 1:** Demographics parameters and serum lipids levels of the PACG and control groups.

	**PACG group**	**Control group**	***T* value**	***P*-value**
No.	320	242		
Age (years)	60.5 ± 11.8	58.9 ± 8.88	1.755	0.08
Gender (Male/Female)	146/174	111/131	0.057	0.95
Diabetes (Yes/No)	27/293	15/227	0.999	0.318
Hypertension (Yes/No)	96/224	61/181	1.573	0.210
SBP (mm Hg)	129.06 ± 12.11	127.23 ± 18.33	1.320	0.188
DBP (mm Hg)	73.41 ± 8.77	72.67 ± 9.86	0.906	0.365
BMI (Kg/m^2^)	22.73 ± 3.4	23.47 ± 5.78	1.019	0.309
IOP (mm Hg)	21.46 ± 11.16	–		
VCDR	0.63 ± 0.28	–		
ACD (cm)	1.93 ± 0.48	–		
CCT (mm)	544.30 ± 40.86	–		
AL (cm)	22.56 ± 1.38	–		
MD (dB)	13.92 ± 8.58	–		
MS (dB)	13.43 ± 8.64	–		
LDL-C (mmol/L)	2.67 ± 0.79	2.51 ± 0.58	2.813	0.005
TRIG (mmol/L)	1.79 ± 1.31	1.51 ± 0.97	2.859	0.004
HDL-C (mmol/L)	1.21 ± 0.34	0.96 ± 0.21	9.764	<0.001
SDLDL-C (mmol/L)	0.34 ± 0.29	0.35 ± 0.18	0.325	0.745
APOA (mmol/L)	1.49 ± 0.31	1.23 ± 0.27	10.32	<0.001
APOB (mmol/L)	0.92 ± 0.23	0.81 ± 0.19	6.358	<0.001
APOE (mmol/L)	39.27 ± 10.5	34.6 ± 9.52	5.42	<0.001
LPa (mmol/L)	189.28 ± 20.1	138.7 ± 16.1	3.125	0.002
CHOL (mmol/L)	4.71 ± 1.01	4.10 ± 0.77	7.682	<0.001

*VCDR, vertical cup/disc ratio; CCT, central corneal thickness; ACD, anterior chamber depth; AL, axial length; MD, mean deviation values for the visual field; MS, mean sensitivity values for the visual field; IOP, intraocular pressure; BMI, body mass index; SBP, systolic blood pressure; DBP, diastolic blood pressure; PACG, primary angle closure glaucoma; HDL-C, high-density lipoprotein cholesterol; LDL-C, low-density lipoprotein cholesterol; SDLDL-C, small dense low-density lipoprotein cholesterol; TRIG, triglyceride; CHOL, cholesterol; APOA, apolipoprotein A; APOB, apolipoprotein B; APOE, apolipoprotein E; Lpa, lipoprotein a*.

### Comparison of Serum Lipid Levels in PACG Subjects in the Sex- and Age-based Subgroups

The PACG subjects were categorized into sex-based female and male subgroups. In both the female and male subgroups, the subjects were further divided into two groups based on age (>60 subgroup, ≤60 subgroup). In the male subgroup ≤60 years, the mean serum TRIG, APOE, and LPa levels were significantly higher (*P* < 0.005) in the PACG group compared to the control group. In the male subgroup >60 years, the mean serum HDL-C and APOE levels were significantly higher in the PACG group compared to the control group (Supplementary Table 2).

In the female subgroup ≤60 years, the mean serum HDL-C, SDLDL-C, APOA, APOB, APOE, and CHOL levels were significantly higher (*P* < 0.005) in the PACG group compared to the control group. In the female subgroup >60 years, the mean serum LDL-C, SDLDL-C, APOA, APOB, APOE, LPa, and CHOL levels were significantly higher (*P* < 0.005) in the PACG group compared to the control group (Supplementary Table 3).

### Association Between Serum Lipids and Ocular Parameters

Pearson correlation analysis was carried out between the serum lipid levels and ocular parameters. Pearson analysis showed that the serum CHOL, HDL-C, LDL-C, APOA, and APOB levels were positively correlated with the mean deviation (MD) level (*r* = 0.247, *P* < 0.001; *r* = 0.202, *P* < 0.001; *r* = 0.229, *P* < 0.001; *r* = 0.218, *P* < 0.001; *r* = 0.175, *P* < 0.05, respectively; Table 2).

**Table 2:** Association between serum lipids and ocular parameters.

	**IOP**	**VCDR**	**AL**	**CCT**	**ACD**	**MD**
LDL-C	*R* = 0.001	*R* = 0.081	*R* = 0.030	*R* = 0.069	*R* = 0.015	*R* = 0.229
	*P* = 0.985	*P* = 0.166	*P* = 0.600	*P* = 0.238	*P* = 0.790	*P* < 0.001
TRIG	*R* = 0.042	*R* = 0.063	*R* = 0.022	*R* = 0.030	*R* = 0.045	*R* = 0.047
	*P* = 0.461	*P* = 0.281	*P* = 0.701	*P* = 0.610	*P* = 0.438	*P* = 0.454
HDL-C	*R* = 0.049	*R* = 0.064	*R* = 0.142	*R* = 0.037	*R* = 0.12	*R* = 0.202
	*P* = 0.389	*P* = 0.274	*P* = 0.014	*P* = 0.534	*P* = 0.843	*P* = 0.001
SDLDL-C	*R* = 0.014	*R* = 0.050	*R* = 0.115	*R* = 0.057	*R* = 0.078	*R* = 0.004
	*P* = 0.799	*P* = 0.392	*P* = 0.047	*P* = 0.331	*P* = 0.181	*P* = 0.948
APOA	*R* = 0.116	*R* = 0.011	*R* = 0.160	*R* = 0.007	*R* = 0.008	*R* = 0.218
	*P* = 0.040	*P* = 0.849	*P* = 0.006	*P* = 0.904	*P* = 0.885	*P* < 0.001
APOB	*R* = 0.031	*R* = 0.036	*R* = 0.032	*R* = 0.006	*R* = 0.004	*R* = 0.175
	*P* = 0.580	*P* = 0.537	*P* = 0.581	*P* = 0.925	*P* = 0.947	*P* = 0.004
APOE	*R* = 0.003	*R* = 0.052	*R* = 0.005	*R* = 0.100	*R* = 0.034	*R* = 0.090
	*P* = 0.957	*P* = 0.378	*P* = 0.931	*P* = 0.089	*P* = 0.556	*P* = 0.148
LPa	*R* = 0.024	*R* = 0.004	*R* = 0.029	*R* = 0.052	*R* = 0.054	*R* = 0.025
	*P* = 0.677	*P* = 0.939	*P* = 0.615	*P* = 0.377	*P* = 0.352	*P* = 0.690
CHOL	*R* = 0.032	*R* = 0.083	*R* = 0.020	*R* = 0.054	*R* = 0.001	*R* = 0.247
	*P* = 0.571	*P* = 0.159	*P* = 0.732	*P* = 0.361	*P* = 0.991	*P* < 0.001

*VCDR, vertical cup/disc ratio; CCT, central corneal thickness; ACD, anterior chamber depth; AL, axial length; MD, mean deviation values for the visual field; IOP, intraocular pressure; HDL-C, high-density lipoprotein cholesterol; LDL-C, low-density lipoprotein cholesterol; SDLDL-C, small dense low-density lipoprotein cholesterol; TRIG, triglyceride; CHOL, cholesterol; APOA, apolipoprotein A; APOB, apolipoprotein B; APOE, apolipoprotein E; Lpa, lipoprotein a*.

### Logistic Regression Analysis of the Association Between Serum Lipid Levels in the PACG and Control Subjects

Logistic regression analyses revealed that serum HDL-C (odds ratio [OR] = 11.01, 95% confidence interval [CI] = 5.616–21.587), SDLDL-C (OR = 1.007, 95% CI = 1.005–1.008), APOA (OR = 13.621, 95% CI = 7.251–25.591), APOB (OR = 2.243, 95% CI = 1.060–4.732), LPa (OR = 0.999, 95% CI = 0.998–1.00), and CHOL levels (OR = 1.131, 95% CI = 1.005–1.326) were associated with PACG after adjusting for age and other demographic parameters (Table 3).

**Table 3:** Logistic regression analysis of the association between serum lipid levels and PACG.

	***B***	***P***	**OR**	**95%CI**
LDL-C	0.285	0.008	1.330	1.079–1.640
TRIG	0.025	0.501	1.025	0.953–1.103
HDL-C	2.399	<0.001	11.01	5.616–21.587
SDLDL-C	0.007	<0.001	1.007	1.005–1.008
APOA	2.612	<0.001	13.621	7.251–25.591
APOB	0.808	0.034	2.243	1.060–4.732
APOE	0.013	0.094	1.104	0.998–1.030
LPa	0.001	0.030	0.999	0.998–1.00
CHOL	0.123	<0.001	1.131	1.005–1.326

*Adjusted for age, gender, BMI, diabetes, hypertension, systolic blood pressure (SBP), diastolic blood pressure (DBP). HDL-C, high-density lipoprotein cholesterol; LDL-C, low-density lipoprotein cholesterol; SDLDL-C, small dense low-density lipoprotein cholesterol; TRIG, triglyceride; CHOL, cholesterol; APOA, apolipoprotein A; APOB, apolipoprotein B; APOE, apolipoprotein E; Lpa, lipoprotein a*.

### Multiple Linear Regressions for Associations Between Lipid Levels and MD in PACG Subjects

Multiple linear regression analysis, adjusting for age, sex, body mass index, systolic blood pressure, diastolic blood pressure, hypertension, diabetes, IOP, central corneal thickness, anterior chamber depth, and axial length was performed to further analyze the association between serum lipid levels and MD (Table 4). There was a statistically correlation between HDL-C levels and MD (*B* = 0.389, *P* = 0.002, 95%CI = −1.249 to −0.624); LDL-C levels and MD (*B* = 0.190, *P* = 0.019, 95%CI = −5.632 to −1.306); and CHOL levels and MD (*B* = 0.364, *P* = 0.027, 95%CI = −7.722 to −1.839).

**Table 4:** Multiple linear regressions for associations between lipid levels in patients with PACG.

	***B***	***P***	**95%CI**
LDL-C	0.190	0.019	−5.632 to −1.306
TRIG	0.052	0.512	−8.175 to 4.139
HDL-C	0.389	0.002	−1.249 to −0.624
SDLDL-C	0.011	0.886	−15.303 to −3.517
APOA	0.089	0.555	−10.313 to 5.552
APOB	0.013	0.931	−10.980 to 10.050
APOE	0.034	0.598	−0.126 to 0.073
LPa	0.057	0.346	−0.007 to 0.002
CHOL	0.364	0.027	−7.722 to −1.839

*Adjusted for age, gender, BMI, diabetes, hypertension, systolic blood pressure, diastolic blood pressure. HDL-C, high-density lipoprotein cholesterol; LDL-C, low-density lipoprotein cholesterol; SDLDL-C, small dense low-density lipoprotein cholesterol; TRIG, triglyceride; CHOL, cholesterol; APOA, apolipoprotein A; APOB, apolipoprotein B; APOE, apolipoprotein E; Lpa, lipoprotein a*.

## Discussion

The following points were found in our research. First, the serum LDL-C, TRIG, HDL-C, APOE, LPa, CHO, APOB, and APOA levels were higher in the PACG patients. Second, the serum CHOL, HDL-C, LDL-C, APOA, and APOB levels were positively correlated with the MD level. Finally, high serum HDL-C, SDLDL-C, APOA, APOB, Lpa, and CHOL levels were associated with PACG. The results of our study indicate that higher serum HDL-C, SDLDL-C, APOA, APOB, and LPa levels are associated with a significantly increased risk of PACG. These findings are consistent with the literature (7, 14).

The pathogenesis of PACG is complex; shallow anterior chamber, short axial length, hyperopia, and east Asian ethnic origin are the main risk factors for primary angle-closure glaucoma (6, 15). Furthermore, abnormal lipid metabolism is an important risk factor for cardiovascular disease and is associated with many eye diseases (16). In addition, a large number of studies have shown that abnormal lipid metabolism hyperlipidemia is associated with glaucoma (8). The study of Edwards et al. (17) indicated that endogenous lipids may be involved in the regulation of IOP homeostasis. Furthermore, Tang et al. (18) showed that TRIG were higher, and was an independent risk factor for POAG. Other studies have also confirmed the association between LDL-C and glaucoma (9, 19). Yilmaz et al. (9) also found increased TRIG and CHOL levels in normal intraocular pressure glaucoma. Lee et al. (20) found that hyperlipidemia had a certain correlation with the occurrence and development of POAG. One study (21) found that glaucoma-like pathological changes occurred in the eyes of rabbits fed a high-fat diet. Other studies (22–24) also found that the lipid levels and types of aqueous humor and trabecular mesh in glaucoma animal models and patients were significantly different from those in the control group, indicating that lipid levels were correlated with glaucoma. Our results also indicate that hyperlipidemia has a certain correlation with the occurrence and severity of PACG.

Currently, the mechanism by which hyperlipidemia increases the risk of glaucoma onset and progression is unclear. Ishikawa et al. (25) showed that high serum lipid levels would increase peripheral venous pressure and blood viscosity, leading to a decrease in blood outflow. Wang et al. (16) found that lipid metabolic abnormalities can change hemodynamics and affect the aqueous humor outflow, which can lead to elevated IOP. Other scholars have also confirmed that hyperlipidemia can increase blood viscosity, in turn, affecting the aqueous humor circulation pathway, leading to an elevated intraocular pressure, which may lead to glaucoma (26, 27). Moreover, increased serum lipid levels might cause degenerative changes in ocular blood vessels, leading to increased production of reactive oxygen species, and thus accelerate the development of glaucoma (24). Kim et al. (28) found that increased LDL-C levels can increase blood viscosity, thereby affecting the microcirculation of the eye, and thus participate in the development of glaucoma.

In this study, the level of LDL-C was higher in older female PACG patients, but not in younger female PACG patients. The reason for this finding may be explained by menopausal status differences. Schaefer et al. (29) reported that increased age was associated with higher plasma LDL-C levels, especially in women. After adjustment for age and BMI, LDL-C level was still significantly higher in older female PACG patients than in younger female PACG patients, indicating a hormonal effect on LDL metabolism.

Moreover, the level of HDL-C was significantly higher in younger female PACG patients, but not in older female PACG patients. From our results, the following two questions arise: first, why is the serum concentration of HDL-C higher in subjects with PACG? Traditionally, HDL-C is considered as protective for cardiovascular disease and also known as “good cholesterol”; Second, what could be the reason behind the difference in the serum levels of HDL-C between younger and older female subjects with PACG when compared with the control groups? Limited data are available in the literature regarding these results. The reasons for these findings are currently not well-understood. Further research is needed to better understand the complex relationship and possible mechanism between serum lipid level and PACG.

Furthermore, significantly higher levels of fewer types of lipids in males compared to female subjects were observed in this study. The explanation for this may be explained by sex differences in obesity, and physical activity (30–32). For example, Saquib et al. (30) reported that the prevalence of both generalized (79 vs. 53%) and central obesity (85 vs. 42%) were significantly higher in women than men. Women reported spending more time watching TV and spending less time walking than men (*p* < 0.05). Qiao et al. (31) reported that women were more likely than men to have diabetes and obesity, and to have higher CHOL, and LDL-C levels.

Our research has certain limitations. First, our study is a cross-sectional, case–control study, and the ability to explore the exact mechanism of the association between serum lipid levels and PACG is still insufficient. This, in turn, limits our ability to make accurate inferences about the causal relationship between serum lipid levels and PACG. Second, the subjects involved in this study were Chinese, which may limit the universality of the results. Finally, we did not consider the treatment of lipid-lowering drugs and how they might affect the test results. Therefore, in the future, multi-center, prospective research with large sample sizes should be carried out.

In conclusion, the results of this study showed that the serum LDL-C, TRIG, HDL-C, APOE, LPa, CHOL, APOB, and APOA levels in PACG patients were significantly higher than those in the control group. Higher serum HDL-C, SDLDL-C, APOA, APOB, and LPa levels were significantly correlated with PACG. Therefore, the exploration of treatment methods from the lipid level will open up a new direction for the treatment of PACG. However, a multi-center, in-depth investigation of clinical studies is needed for further research and demonstration.

## Data Availability Statement

The raw data supporting the conclusions of this article will be made available by the authors, without undue reservation.

## Ethics Statement

The studies involving human participants were reviewed and approved by the Ethics Committee of the Eye and ENT Hospital of Fudan University. The patients/participants provided their written informed consent to participate in this study. Written informed consent was obtained from the individual(s) for the publication of any potentially identifiable images or data included in this article.

## Author Contributions

SL, MS, and WC conceived the study and participated in drafting the final manuscript. SL, YL, JT, MS, and WC analyzed the data and completed the final draft of the manuscript. All authors have read and approved the manuscript.

## Conflict of Interest

The authors declare that the research was conducted in the absence of any commercial or financial relationships that could be construed as a potential conflict of interest.
