# Supplementary material for: Association Between Serum Lipid Levels and Patients With Primary Angle-Closure Glaucoma in China: A Cross Sectional, Case–Control Study
Source: Front Med (Lausanne). 2021 Feb 2;8:618970. doi: 10.3389/fmed.2021.618970 (PMC7884458; doi:10.3389/fmed.2021.618970)
Supplement: Supplementary file 1 [file Data_Sheet_1.docx]

Table S1. Demographics Parameters of the newly diagnosed PACG patients and referral PACG patients

|  | Newly diagnosed | Referral PACG | T value | P value |
| --- | --- | --- | --- | --- |
| Age (years) | 59.96±11.20 | 62.13±11.31 | 1.594 | 0.112 |
| Gender (Male/Female) | 94/108 | 52/66 | 0.183 | 0.669 |
| Diabetes (Yes/No) | 16/186 | 11/107 | 0.189 | 0.663 |
| Hypertension (Yes/No) | 56/146 | 40/78 | 1.353 | 0.245 |
| SBP (mm Hg) | 127.50±17.25 | 127.10±19.17 | 0.175 | 0.861 |
| DBP (mm Hg) | 73.52±10.02 | 72.02±9.68 | 0.587 | 0.205 |
| BMI (Kg/m^2^) | 23.47±3.30 | 23.41±13.65 | 0.037 | 0.971 |
| IOP (mm Hg) | 21.15±10.98 | 21.60±11.93 | 0.324 | 0.746 |
| VCDR | 0.60±0.22 | 0.65±0.29 | 1.545 | 0.123 |
| MD (dB) | 12.51±7.70 | 13.65±8.78 | 1.415 | 0.158 |
| MS (dB) | 14.60±7.77 | 13.77±8.84 | 1.644 | 0.101 |

VCDR: vertical cup/disc ratio, MD: mean deviation values for the visual field, MS: mean sensitivity values for the visual field, IOP: intraocular pressure, BMI: body mass index, SBP: systolic blood pressure, DBP: diastolic blood pressure, PACG primary angle closure glaucoma.

Table S2. Comparison of blood lipid levels in male PACG subjects

|  | PACG | Control | t | p |
| --- | --- | --- | --- | --- |
| Number (≤60/>60) | 63/83 | 46/65 | 0.075 | 0.784 |
| Age (years) | 59.3±12.76 | 57.1±9.98 | 1.55 | 0.122 |
| LDL-C | 2.53±0.71 | 2.43±0.54 | 1.32 | 0.187 |
| ≤60 | 2.51±0.78 | 2.42±0.56 | 0.72 | 0.47 |
| >60 | 2.56±0.65 | 2.45±0.52 | 0.928 | 0.355 |
| TRIG | 1.56±0.96 | 1.12±0.41 | 4.47 | <0.001 |
| ≤60 | 1.57±1.0 | 1.06±0.42 | 3.96 | <0.001 |
| >60 | 1.54±0.94 | 1.23±0.38 | 1.957 | 0.053 |
| HDL-C | 1.09±0.31 | 0.98±0.21 | 3.21 | 0.002 |
| ≤60 | 1.09±0.29 | 1.01±0.21 | 1.84 | 0.066 |
| >60 | 1.09±0.323 | 0.925±1.77 | 2.926 | 0.004 |
| SDLDL-C | 0.52±0.32 | 0.43±0.21 | 2.62 | 0.002 |
| ≤60 | 0.51±0.28 | 0.42±0.21 | 1.91 | 0.058 |
| >60 | 0.545±0.35 | 0.46±0.18 | 1.359 | 0.177 |
| AP0A | 1.34±0.24 | 1.41±0.21 | 2.184 | 0.009 |
| ≤60 | 1.35±0.26 | 1.45±0.22 | 2.39 | 0.018 |
| >60 | 1.33±0.22 | 1.31±0.157 | 0.53 | 0.592 |
| APOB | 0.91±0.21 | 0.91±0.18 | 0.513 | 0.61 |
| ≤60 | 0.89±0.22 | 0.91±0.19 | 0.49 | 0.61 |
| >60 | 0.913±0.21 | 0.933±0.155 | 0.524 | 0.601 |
| APOE | 37.8±9.15 | 33.41±9.71 | 3.738 | <0.001 |
| ≤60 | 36.87±8.13 | 32.8±9.91 | 2.58 | 0.011 |
| >60 | 38.65±9.83 | 34.68±9.28 | 2.09 | 0.038 |
| LPa | 135.7±19.1 | 189.81±22.1 | 1.988 | 0.048 |
| ≤60 | 180.3±24.1 | 109.6±18.2 | 1.96 | <0.05 |
| >60 | 197.19±22.6 | 189.9±20.7 | 0.163 | 0.871 |
| CHOL | 4.51±0.93 | 4.26±0.65 | 2.259 | 0.025 |
| ≤60 | 4.51±0.98 | 4.27±0.67 | 1.61 | 0.109 |
| >60 | 4.5±0.91 | 4.25±0.58 | 1.51 | 0.132 |

HDL-C: high-density lipoprotein cholesterol, LDL-C: low-density lipoprotein cholesterol, SDLDL-C: small dense low-density lipoprotein cholesterol, TRIG: triglyceride, CHOL: cholesterol, APOA: apolipoprotein A, APOB: apolipoprotein B, APOE: apolipoprotein E, Lpa: lipoprotein a.

Table S3. Comparison of blood lipid levels in female PACG subjects

|  | PACG | Control | t | p |
| --- | --- | --- | --- | --- |
| Number (≤60/>60) | 75/99 | 59/72 | 0.114 | 0.736 |
| Age(years) | 61.5±11.0 | 60.5±7.6 | 0.91 | 0.36 |
| LDL-C | 2.79±0.83 | 2.57±0.61 | 2.56 | 0.011 |
| ≤60 | 2.58±0.789 | 2.513±0.60 | 0.616 | 0.539 |
| >60 | 2.95±0.84 | 2.64±0.60 | 2.518 | 0.013 |
| TRIG | 1.99±1.51 | 1.84±1.17 | 0.979 | 0.328 |
| ≤60 | 1.95±1.12 | 1.78±1.18 | 0.708 | 0.481 |
| >60 | 2.026±0.84 | 1.90±1.17 | 0.572 | 0.568 |
| HDL-C | 1.32±0.34 | 0.957±0.22 | 10.58 | <0.001 |
| ≤60 | 1.298±0.34 | 0.93±0.25 | 7.31 | <0.001 |
| >60 | 2.026±0.84 | 1.90±1.17 | 0.572 | 0.568 |
| SDLDL-C | 0.19±0.14 | 0.28±0.12 | 5.68 | <0.001 |
| ≤60 | 0.181±0.13 | 0.26±0.09 | 3.38 | <0.001 |
| >60 | 0.205±0.143 | 0.313±0.148 | 4.578 | <0.001 |
| APOA | 1.63±0.31 | 1.08±0.23 | 16.29 | <0.001 |
| ≤60 | 1.60±0.31 | 1.03±0.258 | 11.62 | <0.001 |
| >60 | 1.645±0.317 | 1.147±0.188 | 11.10 | <0.001 |
| APOB | 0.95±0.24 | 0.72±0.15 | 9.32 | <0.001 |
| ≤60 | 0.896±0.23 | 0.70±0.15 | 5.818 | <0.001 |
| >60 | 0.992±0.23 | 0.75±0.165 | 7.019 | <0.001 |
| APOE | 40.4±11.4 | 35.62±9.27 | 3.947 | <0.001 |
| ≤60 | 39.12±11.02 | 34.54±10.1 | 2.481 | 0.014 |
| >60 | 41.4±10.94 | 36.86±8.12 | 2.828 | 0.005 |
| LPa | 188.78±18.32 | 141.19±12.81 | 2.53 | 0.012 |
| ≤60 | 162.29±16.6 | 142.83±14.8 | 0.741 | 0.461 |
| >60 | 208.84±19.3 | 139.3±10.12 | 2.592 | 0.010 |
| CHOL | 4.87±1.04 | 3.96±0.85 | 8.15 | <0.001 |
| ≤60 | 4.62±1.03 | 3.80±0.88 | 5.119 | <0.001 |
| >60 | 5.068±1.01 | 4.162±0.757 | 6.032 | <0.001 |

HDL-C: high-density lipoprotein cholesterol, LDL-C: low-density lipoprotein cholesterol, SDLDL-C: small dense low-density lipoprotein cholesterol, TRIG: triglyceride, CHOL: cholesterol, APOA: apolipoprotein A, APOB: apolipoprotein B, APOE: apolipoprotein E, Lpa: lipoprotein a.
